# Emodin inhibits LOVO colorectal cancer cell proliferation via the regulation of the Bcl-2/Bax ratio and cytochrome *c*

**DOI:** 10.3892/etm.2014.1900

**Published:** 2014-08-11

**Authors:** LIANG MA, WUSHENG LI

**Affiliations:** Department of Anorectal Surgery, The Affiliated Traditional Chinese Medicine Hospital of Luzhou Medical College, Luzhou, Sichuan 646000, P.R. China

**Keywords:** emodin, cell proliferation, B-cell lymphoma-2, B-cell lymphoma-2 associated X protein, cytochrome *c*

## Abstract

In this study, the effect of emodin and its mechanism of action were investigated in LOVO colorectal cancer cells. Cell growth was determined using a Cell Counting kit-8 assay, and the results demonstrated that emodin significantly inhibited the growth of LOVO cells in a concentration-dependent manner. In order to investigate the anticancer mechanism of emodin, reverse transcription polymerase chain reaction assays were performed to determine the B-cell lymphoma-2 (Bcl-2)/Bcl-2-associated X protein (Bax) expression ratio in LOVO colorectal cancer cells following treatment with emodin. The results showed that emodin induced a significant increase in the Bax expression level and a marked reduction of the Bcl-2 expression level in LOVO cells. In addition, emodin was found to have an inhibitory effect on the mitochondrial membrane potential and the results from the western blot analysis revealed that cytochrome *c* was released from the mitochondria to the cytoplasm. In combination, these results suggest that emodin inhibits cancer cell growth via the regulation of the Bcl-2/Bax ratio and by its effect on the mitochondrial apoptosis pathway.

## Introduction

Colorectal cancer is one of the leading causes of mortality and is a major public health concern worldwide (Guo). The incidence of colorectal cancer is also increasing among the Chinese population (Jin). Chemoprevention is the most promising strategy since other therapies are not effective in controlling gastrointestinal cancers (Andre). However, recent studies have focused on the antitumor properties of natural products since these materials typically have fewer side-effects and are more suitable for long-term use compared with chemically synthesized medicines (16). Emodin, a naturally occurring compound that may be extracted from various Chinese plants, including *Rheum officinale* and *Polygonum cuspidatum* (1), is known for its anticarcinogenic effects that have been demonstrated in a variety of experimental cancer models (2). Pharmacological studies have shown that emodin exhibits anticancer effects in a number of human cancers (3). In addition, previous studies have shown that emodin exerts antiproliferative and apoptosis-inducing effects on cell lines derived from ovarian (4) and lung (5) cancer and leukemia (6). However, at present there is little information demonstrating the possible antiproliferative effect of emodin on colorectal cancer. Therefore, in the present study, the inhibitory effect of emodin on cell proliferation in a colorectal cancer cell line was investigated, as well as the molecular mechanisms involved.

## Materials and methods

### Cell culture and treatment

LOVO colorectal cancer cells were purchased from the American Type Culture Collection (ATCC, Manassas, VA, USA). The cells were cultured as monolayers in RPMI-1640 medium (Gibco-BRL, Gaithersburg, MD, USA) supplemented with 10% fetal bovine serum (Gibco-BRL, São Paulo, Brazil) and 1% penicillin/streptomycin. The cells were grown at 37°C in a humidified atmosphere containing 5% CO_2_. Emodin was purchased from Sigma-Aldrich (St. Louis, MO, USA) and was freshly diluted to concentrations of 0, 10, 20 and 40 μmol/l with dimethyl sulfoxide (DMSO). The final concentration of DMSO was <0.1%. The cells were treated with different concentrations of emodin for 24 h and then analyzed.

### Cell viability assay

Cell viability was determined using Cell-Counting kit-8 (CCK-8, Dojindo Laboratories, Tokyo, Japan). Cells were seeded into a 96-well plate at a density of 5×10^5^ cells/well and cultured for 24 h. Emodin was then added to the wells with final concentrations of 0, 10, 20 and 40 μmol/l; DMSO alone was used for the control group. After 24 h, CCK-8 solution was added (10 μl to each well containing 100 μl medium). The plates were incubated at 37°C for 4 h, and the absorbance at 450 nm was then measured using a Benchmark Plus microplate reader (Bio-Rad, Hercules, CA, USA). All experiments were performed in triplicate.

### Reverse transcription polymerase chain reaction (RT-PCR)

Total RNA was isolated from cells using TRIzol^®^ reagent (Invitrogen Life Technologies, Carlsbad, CA, USA) and quantified using UV absorption at 260 and 280 nm. RT-PCR was performed in accordance with the reverse-transcription PCR (RT-PCR) reaction kit (GIBCO, Grand Island, NY, USA) instructions. RT-PCR primers were designed as follows: B-cell lymphoma-2 (Bcl-2) forward, 5′-CTTTTGCTGTGGGGT TTTGT-3′ and reverse, 5′-GTCATTCTGGCCTCTCTTGC-3′; Bcl-2-associated X protein (Bax) forward, 5′-GGAGCT GCAGAGGATGATTG-3′ and reverse, 5′-CCTCCCAGA AAAATGCCATA-3′. The RT-PCR process was performed as follows: Denaturation for 1 min at 93°C, followed by 31, 33 and 32 cycles of denaturing for 30 sec at 94°C, annealing for 1 min at 57°C, 60°C and 57°C for GAPDH, Bcl-2 and Bax, respectively, and extension for 2 min at 72°C. The amplified products were visualized using 1.5% agarose gel electrophoresis, stained with ethidium bromide and images were then captured under ultraviolet light. Densitometric analysis of three different observations was performed using Quantity One Software (Bio-Rad). The quantity of each transcript was normalized against GAPDH.

### Western blot analysis

The cells were harvested at the indicated times, and lysed with lysis buffer [50 mM Tris Cl, (pH 7.8), 150 mM NaCl, 1% NP_4_0, 0.1% SDS, 1 mM phenylmethylsulfonyl fluoride]. The total protein (50 μg per well) was separated by 10% SDS-PAGE gels, and electrophoretically transferred onto nitrocellulose membranes. Following blocking for 1 h with 5% skimmed milk in Tris-buffered saline (TBS) buffer (10 mM Tris and 150 mM NaCl), the membrane was washed 3 times for 18 min each with TBST buffer (10 mM Tris, 150 mM NaCl and 0.1% Tween-20). Immunoreactive bands were visualized using horseradish peroxidase-conjugated secondary antibody (1:5,000 dilution; Beyotime Institute of Biotechnology, Haimen, Jiangsu, China) and an enhanced chemiluminescene western blotting detection kit (Amersham, Little Chalfont, Buckinghamshire, UK). The bands were visualized using the enhanced chemilluminescence system, and the band density was determined by Image J software (National Institutes of Health, Bethestha, MD, USA. The antibodies were all purchased from Beyotime Institute of Biotechnology.

### Mitochondrial membrane potential assay

Mitochondrial potential was analyzed using the fluorescent potentiometric dye JC-1 as described previously (7). Briefly, following 24 h of treatment with 0–40 μmol/l emodin, cells were harvested, washed twice with phosphate-buffered saline (PBS) and centrifuged for 8 min at 450 × g at room temperature. The cells were then suspended with JC-1 (5 μg/ml) in serum-free RPMI-1640 and incubated for 15 min at 37°C. Following staining, the cells were collected at room temperature and washed three times with pre-warmed PBS. The cell pellet was then re-suspended in 1 ml PBS. Fluorescence emitted by JC-1 was quantified using a fluorescence plate reader (Molecular Devices, Sunnyvale, CA, USA) at 37°C. The fluorescence of the JC-1 monomer was measured at 485 nm excitation wavelength/530 nm emission wavelength. The fluorescence of the JC-1 aggregate was measured at 535 nm excitation wavelength/590 nm emission wavelength. For each experiment, the ratios of JC-1 aggregate to JC-1 monomer were normalized against untreated controls; the values therefore represent a percentage of the mitochondrial function in untreated cells.

### Analysis of cytochrome c release

Cytochrome *c* release from the mitochondria was evaluated by western blot analysis of cytosolic protein samples (8,9). Cells were harvested following treatment with emodin for 24 h and mixed with 100 μl cold lysis buffer [50 mmol/l Tris-HCl (pH 7.4), 1 mmol/l NaF, 150 mmol/l NaCl, 1 mmol/l ethylene glycol tetraacetic acid, 1 mmol/l phenylmethylsulfonyl fluoride (PMSF), 1% (v/v) Nonidet P-40 (NP-40) and 10 μg/ml leupeptin], followed by centrifugation at 10,000 × g for 30 min at 4°C. The supernatant was centrifuged two more times under the same conditions to remove the nuclear debris. The cytosolic extracts were then used for western blot analysis to analyze the level of cytochrome *c* release. Western blot analysis was subsequently performed as described above.

### Statistical analysis

Data are expressed as the means ± standard deviation of three assays. The statistical analysis was performed using a one-way analysis of variance. A P-value of <0.05 was considered to indicate a statistically significant difference. All statistical analyses were performed using SPSS 13.0 software (SPSS, Inc., Chicago, IL, USA).

## Results

### Effect of emodin on cell viability

**Figure 1:**
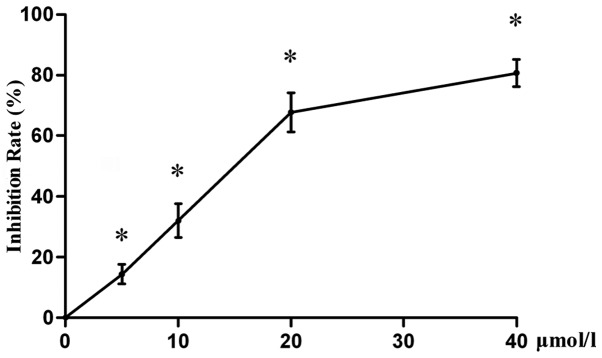
Inhibitory effect of emodin on LOVO colorectal cancer cells. Cells were treated with 0, 5, 10, 20 and 40 μmol/l emodin for 24 h. Cell growth was measured using a Cell Counting kit-8 assay. Data are expressed as mean ± standard deviation of three independent experiments. ^*^P<0.05, compared with the control group (0 μmol/l).

In order to investigate the antiproliferative effect of emodin on LOVO cells *in vitro*, cell proliferation was measured using the CCK-8 assay. The results of the present study (Fig. 1) demonstrated that emodin inhibits cell proliferation in a concentration-dependent manner, compared with that of vehicle-treated controls (P<0.05).

### Effect of emodin on Bcl-2 and Bax expression in LOVO cells

**Figure 2:**
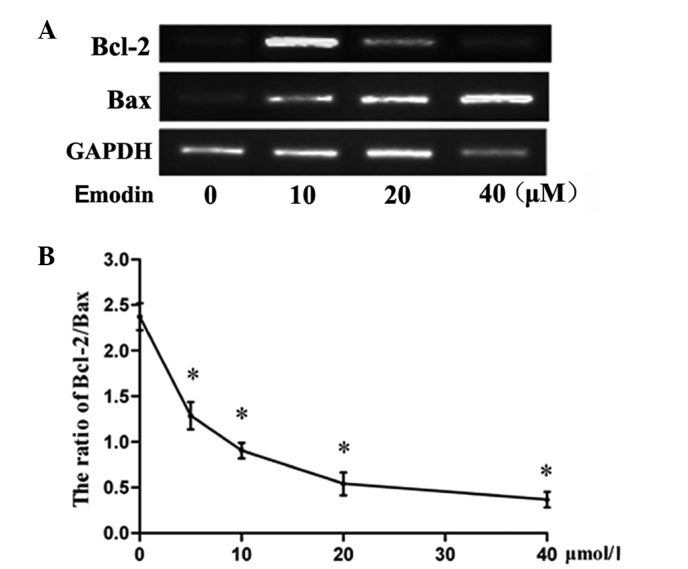
Effect of emodin on Bcl-2 and Bax expression in LOVO cells. (A) Cells were pretreated with emodin (0–40 μM) for 24 h. Following treatment, the expression of Bcl-2 and Bax was determined by reverse transcriptase polymerase chain reaction. (B) The ratio of Bcl-2/Bax decreased in a concentration-dependent manner. ^*^P<0.05 compared with the control group (0 μmol/l). Data are presented as the mean ± standard deviation of three independent experiments. Bcl-2, B-cell lymphoma-2; Bax, Bcl-2-associated X protein.

RT-PCR was performed to determined the Bcl-2 and Bax expression levels of the cells. It was found that emodin upregulated the expression of Bax in the treatment groups compared with that in the control group (P<0.05), whereas it inhibited the expression of Bcl-2 (Fig. 2). Furthermore, significant differences in the Bax/Bcl-2 ratio between the treatment groups and the control cells were observed (Fig. 2).

### Effect of emodin on mitochondrial membrane potential and release of cytochrome c

**Figure 3:**
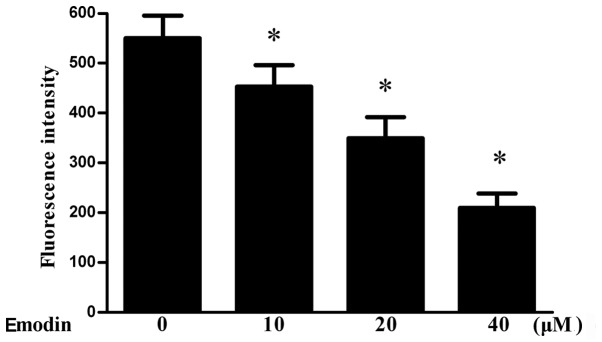
Emodin inhibits mitochondrial membrane potential in LOVO colorectal cancer cells. LOVO cells were treated with emodin (0–40 μM) for 24 h, and the mitochondrial membrane potential was then assessed using the fluorescent potentiometric dye JC-1. ^*^P<0.05, compared with the control group (0 μM). Data are presented as the mean ± standard deviation of three independent experiments.

**Figure 4:**
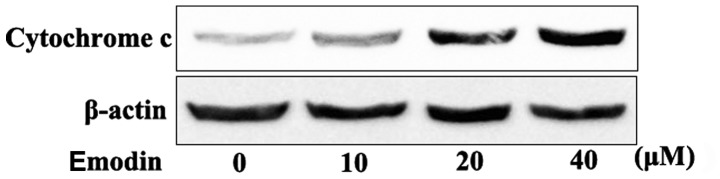
Effect of emodin on cytochrome *c* release. LOVO cells were treated with indicated concentrations of emodin (0–40 μM) for 24 h. The cytochrome c content in the cytosolic fraction was then assayed by western blot analysis.

In order to determine if treatment with emodin decreases the mitochondrial membrane potential, cells were treated with different concentrations of emodin for 24 h prior to measurement of the mitochondrial membrane potential. The results indicated that the mitochondrial membrane potential was significantly decreased by emodin (Fig. 3). In addition, it was found that the release of cytochrome *c* was significantly increased in cells incubated with 10–40 μmol/l emodin (Fig. 4).

## Discussion

Emodin (1,3,8-trihydroxy-6-methylanthraquinone) is a naturally occurring anthraquinone present in rhubarb and numerous other plants. As well as being used as a laxative, emodin has a number of other biological effects, including antibacterial, antiviral, anti-inflammatory and anticancer effects (10). However, at present, there is little evidence of the possible effects of emodin on proliferation in colorectal cancer. Therefore, in the present study, the molecular mechanism involved in the inhibitory effect of emodin on cell proliferation was investigated.

The inhibitory effect of emodin on LOVO colorectal cell proliferation was investigated, as well as the possible underlying mechanisms. It was observed that emodin inhibited LOVO cell proliferation, which is in accordance with a previous study (11).

Bcl-2 family proteins function through different pathways in the regulation of cell apoptosis (12). The Bcl-2 family primarily affects the mitochondrial pathways (13). Bcl-2 and its homologs prevent mitochondrial membrane disruption and the release of cytochrome *c* and other pro-apoptotic factors, while Bax promotes these events. The expression of these two opposing proteins may, in part, determine the fate of cells. The ratio of Bcl-2/Bax is usually regarded as a criterion for programmed cell death (14). The results from the present study demonstrate that emodin upregulates the expression of Bax, but inhibits the expression of Bcl-2. It was also found that there were significant differences in the Bax/Bcl-2 ratio between the treatment and the control groups. These data are consistent with emodin-inhibited cancer cell growth being associated with the balance of Bcl-2/Bax.

In addition, the mitochondrial pathway is a major apoptosis signaling pathway. Numerous studies have shown that the mitochondrial membrane potential stimulates the mitochondrial membrane to open, resulting in the release of cytochrome *c* into the cytoplasm, activation of the caspase pathway and degradation of important intracellular proteins, and consequently the induction of apoptosis (15). In the present study, it was found that the mitochondrial membrane potential was significantly decreased by emodin in a concentration- and time-dependent manner. In addition, the results from the western blot analysis revealed that cytochrome *c* was released from the mitochondria into the cytoplasm, suggesting that the mitochondrial apoptosis pathway is involved in emodin-induced cell line apoptosis. These results are in accordance with a previous study by Wang *et al* (16), which demonstrated that emodin inhibited HeLa proliferation by the induction of apoptosis through the intrinsic mitochondrial pathway.

In conclusion, the results from the present study suggest that emodin inhibits cancer cell growth via the regulation of Bcl-2/Bax and that the mitochondrial apoptosis pathway may be involved.
